# Supplementary material for: Three new species of Microlaimus (Nematoda: Microlaimidae) from the South Atlantic
Source: PeerJ. 2022 Mar 9;10:e12734. doi: 10.7717/peerj.12734 (PMC8917802; doi:10.7717/peerj.12734)
Supplement: Supplemental Information 1 [file peerj-10-12734-s001.docx]

List of valid *Microlaimus* species after Lorenzen (1994), Kovalyev & Tchesunov (2005) Leduc (2016), Shi e Xu (2016), Bezerra *et al.* (2021)

1. *Microlaimus acanthus* (Jayasree & Warwick, 1977) Kovalyev & Tchesunov, 2005

Syn. *Paramicrolaimus* *acanthus* Jayasree & Warwick, 1977

*Calomicrolaimus acanthus* (Jayasree & Warwick, 1977) Jensen, 1978

2. *Microlaimus acicularis* Lorenzen, 1976

3. *Microlaimus acinaces* Warwick & Platt, 1973

4. *Microlaimus acuticaudatus* Stekhoven & De Coninck, 1933

5. *Microlaimus aequisetosus* Blome, 1982

6. *Microlaimus affinis* Gerlach, 1958

7. *Microlaimus africanensis* Furstenberg & Vincx, 1992

8. *Microlaimus alexandri* **sp. n.**

9. *Microlaimus amphidius* Kamran, Nasira & Shahina 2009

10. *Microlaimus annelisae* Jensen, 1976

11. *Microlaimus arenarius* (Blome, 1982) Kovalyev & Tchenusov, 2005

Syn. *Calomicrolaimus arenarius* Blome, 1982

12. *Microlaimus arenicola* Schulz, 1938

13. *Microlaimus bathyalis* (Kovalyev & Miljutina, 2009) Tchesunov, 2014

Syn. *Aponema bathyalis* kovalyev & Miljutina, 2009

14. *Microlaimus borealis* Steiner, 1916

15. *Microlaimus campiensis* **sp. n.**

16. *Microlaimus capillaris* Gerlach, 1957

17. *Microlaimus* *capitatus* Gagarin, 2020

18. *Microlaimus clancularius* Bussau, 1993

19. *Microlaimus cochleatus* Wieser, 1959

20. *Microlaimus compridus* Gerlach, 1956

Syn. *Calomicrolaimus compridus* (Gerlach, 1956) Gourbault & Vincx, 1987

21. *Microlaimus conothelis* (Lorenzen, 1973) ) Jensen, 1978

Syn. *Paramicrolaimus conothelis* Lorenzen, 1973

22. *Microlaimus copulatus* Jensen, 1988

23. *Microlaimus criminalis* Rieger & Ott, 1971

24. *Microlaimus cyatholaimoides* De Man, 1922

25. *Microlaimus decoratus* Pastor de Ward, 1989

26. *Microlaimus dimorphus* Chitwood, 1937

27. *Microlaimus discolensis* Bussau, 1993

28. *Microlaimus dixiei* Wieser, 1959

29. *Microlaimus donsi* Allgén, 1935

30. *Microlaimus falciferus* Leduc & Wharton, 2008

31. *Microlaimus falklandiae* Allgen, 1959

32. *Microlaimus formosus* Gerlach, 1957

Syn. *Calomicrolaimus formosus* (Gerlach, 1957) Jensen, 1978

33. *Microlaimus gerlachi* Wieser, 1954

34. *Microlaimus globiceps* De Man, 1880

35. *Microlaimus honestoides* Meyl, 1954

36. *Microlaimus honestus* De Man, 1922

Syn. *Calomicrolaimus honestus* (De Man, 1922) Jensen, 1978

37. *Microlaimus inermis* Ditlevsen, 1923

38. *Microlaimus karachiensis* Kamran, Nasira & Shahina 2009

39. *Microlaimus kaurii* Wieser, 1954

40. *Microlaimus korari* Leduc, 2016

41. *Microlaimus latilaimus* Allgen, 1959

42. *Microlaimus lepturus* De Cillis & Odorato, 1917

43. *Microlaimus limnophilus* Turpeenniemi, 1997

44. *Microlaimus longispiculum* Timm, 1961

Syn. *Molgolaimus longispiculum* Timm, 1961 (Jensen, 1978)

45. *Microlaimus lunatus* (Wieser & Hopper, 1967) Jensen, 1978

Syn. *Paramicrolaimus lunatus* Wieser & Hopper, 1967

*Microlaimus luneatus* (Wieser & Hopper, 1967) Jensen, 1978

46. *Microlaimus macrocirculus* Gerlach, 1950

47. *Microlaimus macrolaimus* Allgen, 1947

48. *Microlaimus marinus* (Schulz, 1932)

49. *Microlaimus martinezi* (Miljutin & Miljutina, 2009)

Syn. *Aponema martinezi* Miljutin & Miljutina, 2009

50. *Microlaimus microseta* Gerlach, 1953

Syn. *Calomicrolaimus microseta* (Gerlach, 1953) Jensen, 1978

51. *Microlaimus minutissima* (Kovalyev & Miljutina, 2009)

Syn. *Aponema minutissima* kovalyev & Miljutina, 2009

52. *Microlaimus minutus* Muthumbi & Vincx, 1999

53. *Microlaimus monstrosus* Gerlach, 1953

Syn. *Calomicrolaimus monstrosus* (Gerlach, 1953) Vincx, 1981

*Microlaimus conspicuus* Lorenzen, 1973

54. *Microlaimus naidinae* Tchesunov, 1978

55. *Microlaimus nanus* Blome, 1982

56. *Microlaimus nympha* (Bussau, 1993)

Syn. *Aponema nympha* Bussau, 1993

57. *Microlaimus oblongilaimus* Gerlach, 1955

58. *Microlaimus orientalis* Gagarin & Nguyen Vu Thanh, 2011

59. *Microlaimus ostracion* Schuurmans-Stekhoven, 1935

60. *Microlaimus papillatus* Gerlach, 1956

61. *Microlaimus papilliferus* Allgen, 1959

62. *Microlaimus paraborealis* Allgén, 1940

63. *Microlaimus paraconothelis* Kovalyev & Tchesunov, 2005

64. *Microlaimus paraglobiceps* Revkova, 2017

65. *Microlaimus parahonestus* Gerlach, 1950

Syn. *Calomicrolaimus parahonestus* (Gerlach, 1950) Jensen, 1978

66. *Microlaimus parviporosus* Miljutin & Miljutina, 2009

67. *Microlaimus pinguis* Wieser, 1954

68. *Microlaimus ponticus* Sergeeva, 1976

69. *Microlaimus porosus* Bussau, 1993

70. *Microlaimus punctulatus* Gerlach, 1950

71. *Microlaimus pygmaeus* Meyl, 1954

72. *Microlaimus pwani* Muthumbi & Vincx, 1999

73. *Microlaimus robustidens* Schuurmans-Stekhoven & De Coninck, 1933

74. *Microlaimus sensus* Wieser, 1954

75. *Microlaimus sergeevae* Revkova, 2020

76. *Microlaimus setosus* Hoeppli, 1926

Syn. *Microlaimus* (*Microlaimoides*) *setosus* Hoeppli, 1926

77. *Microlaimus sicarius* Wieser, 1954

78. *Microlaimus spinosus* Gerlach, 1957

Syn. *Calomicrolaimus spinosus* (Gerlach, 1957) Jensen, 1978

79. *Microlaimus tenuicollis* Gerlach, 1952

Syn. *Calomicrolaimus tenuicollis* (Gerlach, 1952) Jensen, 1978

80. *Microlaimus tenuispiculum* De Man, 1922

Syn. *Molgolaimus tenuispiculum* De Man, 1922 (Jensen, 1978)

*Molgolaimus* *demani* Jensen, 1978

81. *Microlaimus texianus* Chitwood, 1951

82. *Microlaimus undulatus* Gerlach, 1953

83. *Microlaimus validus* Gagarin & Nguyen Dinh Tu 2014

84. *Microlaimus vitorius* **sp. n.**

85. *Microlaimus westindicus* (Kovalyev & Miljutina, 2009) Tchenusov, 2014

Syn. *Aponema westindicus* Kovalyev & Miljutina, 2009

*Aponema westindicum* Kovalyev & Miljutina, 2009

86. *Microlaimus wieseri* Hopper, 1961

Syn. *Bolbolaimus wieseri* (Hopper, 1961) Jensen, 1978

87. *Microlaimus zosterae* Allgen, 1930
